# Ultrasound-Assisted Deep Eutectic Solvent-Based Green Extraction of Flavonoids from Honeysuckle: Optimization and Mechanistic Insights into α-Amylase Inhibition

**DOI:** 10.3390/foods15010010

**Published:** 2025-12-19

**Authors:** Hong Zong, Guangfan Qu, Feiyan Yang, Fanyu Ye, Yanfeng Liu, Xiang Xu, Xudong He, Qingling Lu, Shuguo Sun

**Affiliations:** 1 National Engineering Laboratory for Deep Process of Rice and Byproducts, Hunan Key Laboratory of Grain-Oil Deep Process and Quality Control, Hunan Key Laboratory of Forestry Edible Resources Safety and Processing, College of Food Science and Engineering, Central South University of Forestry and Technology, Changsha 410004, China; zongh2000@163.com (H.Z.);; 2 Key Laboratory of Industrial Biotechnology, Ministry of Education, School of Biotechnology, Jiangnan University, 1800 Lihu Rd, Wuxi 214122, China; 3 State Key Laboratory of Food Science and Technology, Nanchang University, Nanchang 330047, China

**Keywords:** deep eutectic solvents, honeysuckle, ultrasound-assisted, flavonoid, α-amylase

## Abstract

This study employed deep eutectic solvents (DES) combined with ultrasonic-assisted extraction technology to green and efficiently extract flavonoids from honeysuckle, and systematically evaluated its inhibitory mechanism on α-amylase (α-AMY). Through comparative screening of six DES systems and traditional solvents, DES-4 (Choline chloride–propylene glycol) was identified as the optimal extraction solvent. After single-factor and response surface optimization, the yield of honeysuckle flavonoids (HF) was significantly increased to 9.12 ± 0.08% under the conditions of ultrasonic power 300 W, solid–liquid ratio 1:32 (g/mL), and extraction time 60 min. HPLC-MS analysis revealed that luteolin (4.59 ± 0.09 mg/g) and quercetin (3.05 ± 0.02 mg/g) were the main active components, and they exhibited strong antioxidant activity. Enzyme kinetics and Lineweaver–Burk analysis indicated that the inhibition type of HF on α-AMY was reversible mixed inhibition. Fluorescence spectroscopy, thermodynamic analysis, and molecular docking results further revealed that HF primarily bound to α-AMY through hydrogen bonds and van der Waals forces (ΔH = −63.80 kJ/mol, ΔS = −0.19 J/mol·K), causing static fluorescence quenching and altering its hydrophobic microenvironment and spatial conformation. This study aims to provide new theoretical basis for the green and efficient extraction of HF and its development and application in functional foods and natural medicines.

## 1. Introduction

Honeysuckle (*Lonicera japonica Thunberg*), belonging to the Caprifoliaceae family, was mentioned in Li Shizhen’s “Ben Cao Gang Mu” (A.D. 1552–1578) and is a traditional medicinal and edible plant widely cultivated in East Asian countries such as China, Japan, and South Korea [1]. Honeysuckle contains a variety of bioactive components, such as flavonoids, phenolic acids and saponin compounds [2]. Modern pharmacological studies have shown that honeysuckle flavonoids (HF) have functions such as antioxidant, anti-inflammatory, anti-cancer, liver protection and hypoglycemic effects [3]. Hence, HF has great application potential in the fields of health foods, functional foods, and pharmaceuticals.

Deep eutectic solvents (DESs) have attracted considerable attention in recent years due to their unique characteristics. These solvents are formed by mixing specific hydrogen bond donors (HBDs) and hydrogen bond acceptors (HBAs) and heating the mixture to generate a eutectic liquid through hydrogen-bond interactions. HBAs are commonly represented by quaternary ammonium salts, including choline chloride (Chcl), and the HBDs encompass a range of compounds such as amines, carboxylic acids, and alcohols. Generally, HBDs exist in both solid and liquid forms at room temperature. Heating the mixture of HBDs and HBAs results in the formation of a DES system. DESs present several advantages over traditional solvents, including economic viability, non-toxicity, non-flammability, recyclability, biodegradability, low cost, and environmental friendliness [4]. They also exhibit excellent solubility and extraction properties for organic compounds, making them a promising alternative to conventional solvents in extraction processes. Despite their advantages, DESs also have some limitations. In particular, their inherently high viscosity can hinder solvent penetration into plant cell walls and restrict the diffusion of bioactive compounds, ultimately compromising extraction efficiency. To overcome this issue, the viscosity of DESs is typically modulated by adding a suitable amount of water, thereby facilitating mass transfer and enhancing overall extraction performance [5].

Flavonoids can be extracted from plant materials using several techniques. Traditional methods include maceration, percolation, and Soxhlet extraction [6]. Compared with these conventional approaches, ultrasound-assisted extraction (UAE) has gradually become an effective method for recovering natural ingredients. However, traditional extraction methods often require long processing times and large amounts of organic solvents, which can result in low overall extraction efficiency [7]. This technique primarily exploits the cavitation and thermal effects of ultrasound to enhance material processing, effectively disrupting cell wall structures and promoting molecular diffusion, thereby facilitating the full release of intracellular bioactive compounds. Owing to its short extraction time and high extraction efficiency, this method is widely used in natural product extraction [8]. Studies have shown that ultrasound-assisted extraction can accelerate the extraction of flavonoids while reducing solvent consumption, thus yielding products with higher purity, stronger bioactivity, and increased overall yield. It should be noted, however, that UAE also has certain limitations; for example, the local high temperatures, high pressures, and free radicals generated during ultrasonication may lead to the degradation of thermolabile or structurally unstable components and consequently compromise product quality, making the optimization of process parameters particularly important [9]. He et al. employed an ultrasound-assisted DES extraction technique to efficiently and sustainably extract natural flavonoids from *Fructus aurantii*, they discovered that the use of DES yielded a higher extraction efficiency than conventional solvents, including 95% ethanol, methyl alcohol, and water [10]. Another study show that ultrasonic-assisted DES can efficiently extract flavonoids from *Taro leaf* and show strong antioxidant activity [11].

To the best of our knowledge, although DES–UAE technology has been widely applied for the extraction of active components from various plant matrices, systematic research on its application to HF extraction and on the associated α-amylase (α-AMY) inhibitory activity and potential hypoglycemic mechanisms has not yet been reported. In this study, honeysuckle was used as the raw material, and the DES-UAE process for optimizing the extraction of flavonoids was developed, with the extraction parameters optimized to enhance the extraction rate of flavonoids. Subsequently, the main components were identified by high-performance liquid chromatography–mass spectrometry (HPLC-MS), and their antioxidant activities were evaluated. Finally, the inhibitory mechanism of HF on α-AMY was comprehensively evaluated through in vitro enzyme inhibition assays, kinetic modeling, fluorescence quenching spectroscopy and molecular docking simulations. This study aims to provide a theoretical basis and technical support for the efficient extraction of HF and for elucidating its potential hypoglycemic mechanism.

## 2. Materials and Methods

### 2.1. Materials

Honeysuckle was obtained from Xiawei Biotechnology Co., Ltd., (Changsha, China). Choline chloride (≥98%), citric acid (≥99.5%), malic acid (≥98%), lactic acid (≥98%), propylene glycol (AR, 98%), ethylene glycol (AR, 98%), fructose (≥98%), α-amylase (BR), HPLC-grade solvents and analytical standards were supplied by Paisono Biotechnology Co., Ltd. (Shanghai, China).

### 2.2. Preparation and Characterization of the Properties of DESs

HBA and HBD were mixed at a molar ratio of 1:2, and a specific amount of water (20/40%) was added to decrease the viscosity. The mixture was sealed and stirred in a water bath at 80 °C for one hour until a transparent liquid was obtained, which was subsequently stored at room temperature. The various Deep Eutectic Solvent (DES) codes assigned were 1–6 [12]. The viscosity was determined using a rheometer (DHR-2, Waters, Milford, MA, USA), while the density was measured with slight adjustments following the methodology described by Silva et al. [13] and three parallel tests were conducted (Table 1).

FT-IR (Shimadzu Corp., Kyoto, Japan) was used to examine the ligand, donor and eutectic solvent. KBr was dried at 105 °C for 4–5 h. The sample was ground with KBr powder at a ratio of 1:100 (*w*/*w*). The fine powder was pressed into transparent sheets for detection by the transmission method.

### 2.3. Extraction and Determination of Flavonoids in Honeysuckle

Initially, the pulverized and sieved honeysuckle powder was treated with petroleum ether to remove its oil content via Soxhlet extraction. After the petroleum ether was evaporated, 1 g of the defatted honeysuckle powder was accurately weighed and transferred into a 100 mL Erlenmeyer flask. A DES was then added at a solid–liquid ratio of 1:30 (g raw material/mL DES), followed by ultrasonic extraction and subsequent filtration. The resulting supernatant was collected for further analysis. All procedures were performed in triplicate. A 1 mL aliquot of the extract was transferred into a 10 mL volumetric flask, and the absorbance at 510 nm was measured using the NaNO_2_-Al(NO_3_)_3_-NaOH method. The calculation formula is provided as follows Equation (1):

(1) $$\mathrm{Flavonoid}\;\mathrm{yield}\;Z\;(\%)=\frac{c\;\times \;V\;\times \;N}{m\;\times \;1000}\times 100\%$$

where Z represents the overall extraction efficiency of HF (%); c denotes the mass concentration of total flavonoids (g/L); V indicates the liquid volume of the sample (mL); N refers to the dilution ratio; and m signifies the mass of honeysuckle powder (g).

### 2.4. Optimization of Total Flavonoids Extraction Process of Honeysuckle

#### 2.4.1. Single Factor Test

The effects of various factors on the extraction yield of total flavonoids from honeysuckle were investigated, including the ultrasonic power (100, 200, 300, 400, 500 W), liquid-to-material ratio (1:10, 20, 30, 40, 50 g/mL), extract temperature (20, 35, 50, 65, 80 °C), water content (20, 30, 40, 50, 60%) and extract time (15, 30,45, 60, 80 min). These parameters were systematically optimized to achieve the maximum extraction yield of total flavonoids.

#### 2.4.2. Response Surface Methodology (RSM) Optimization

Based on single factor test, ultrasonic power (A), solid–liquid ratio (B), and water content (C) were taken as investigation variables, and the flavonoids yield y of honeysuckle was taken as response value, with −1, 0 and 1 representing variable levels. According to Box–Behnken Design principle, the tests were arranged by design-Expert 13.0. The factor level coding table and test results were shown in Table S1.

### 2.5. HPLC-MS Analysis

The experiment was conducted using the method of Wang et al., with slight modifications [14]. HPLC-MS targeted analysis was performed on the flavonoids extracted from Honeysuckle by DES under the optimal technological conditions. 

### 2.6. Determination of Scavenging Ability of DPPH and ABTS^+^ Free Radicals

Refer to the method described by Qu et al. [15] and modify it appropriately. Briefly, HF extracts obtained with various solvents were mixed with a 0.001 mmol/mL DPPH solution. After incubating the mixture in the dark for 30 min, the absorbance was measured at 517 nm. The clearance rate is shown in Equation (2):

(2) $$\mathrm{Removal}\;\mathrm{activity}\;(\%)=1-\frac{\left(A_{0}\;-{\;A}_{1}\;\right)}{A_{2}\;}\times 100\%$$

where A_0_: sample absorbance, A_1_: absorbance of the control group, A_2_ corresponds to the blank.

The experimental procedure for scavenging ABTS^+^ free radicals is analogous, first, prepare an ABTS^+^ working solution (ABTS and potassium persulfate solution) with an absorbance of approximately 0.7. Then, add the HF extracts from different solvents to the ABTS^+^ solution. After waiting for 10 min, measure the absorbance value at 734 nm. The calculation formula is the same as Equation (2).

### 2.7. Scanning Electron Microscope (SEM)

In order to further understand the effects of different solvents and methods on the extraction effectiveness of HF, a scanning electron microscope (JSM-6380LV, JEOL Ltd., Tokyo, Japan) was employed to examine the microstructural characteristics of honeysuckle powder prior to and following extraction. The microstructures of the original dried honeysuckle powder were compared with those of the honeysuckle powder extracted by three different extraction methods: ethanol water bath, ethanol-UAE, and DES-4-UAE, the parameters were configured with an acceleration voltage of 5 kV, a working distance of 10 mm, a beam spot size of 30, and the secondary electron imaging mode, to acquire SEM images at various magnifications.

### 2.8. α-AMY Inhibitory Activity

The assessment of α-AMY inhibitory activity was slightly modified from the methodology established by Lordan et al. [16]. Mix 50 μL of HF at different concentrations with a certain volume of α-AMY and PBS. Incubate at 37 °C for 10 min, then add 500 μL of soluble starch and incubate for another 10 min. Finally, add 400 μL of DNS reagent for color development. The calculation of the inhibition rate is shown in Equation (3):

(3) $$\mathrm{Inhibition}\;\mathrm{rate}\;(\%)=[1\;-\;\frac{{\mathrm{OD}}_{A}-{\mathrm{OD}}_{a}}{{\mathrm{OD}}_{B}-{\mathrm{OD}}_{b}}]\;\times \;100\%$$

where OD_A_ and OD_B_ are the absorbance values of the reaction mixtures with HF (sample) and without HF (control), respectively, and OD_a_ and OD_b_ are the absorbance values of their corresponding blanks.

### 2.9. Inhibitory Kinetic Analysis

The experimental method was based on Shen et al. [17] with minor modifications. Briefly, different inhibitor concentrations (0, 0.2, 0.4, 0.6 mg/mL) and α-AMY concentrations (0.1–0.9 mg/mL) were used in the experiment. For the detailed method, refer to Section 2.8. The reaction was carried out in a 96-well plate, and the absorbance was measured at a wavelength of 660 nm. The initial reaction rate (V) was calculated and the curve of the enzymatic reaction rate relationship was plotted. Then, the Lineweaver–Burk double reciprocal diagram and Dixon diagram were constructed under different substrate concentrations, and the types and dynamic characteristics of HF inhibition on α-AMY were analyzed. See Formulas (4) and (5) for drawing and parameter calculation.

(4) $$\frac{1}{V_{0}}=\frac{K_{m}}{V_{m}\left[S\right]}+\frac{1}{V_{m}}$$

(5) $$V_{m}=\frac{V_{m}\left[S\right]}{\left[S\right]\;\left(1+\frac{1}{K_{\mathrm{IS}}}\right)+{\;K}_{m}\;\left(1+\frac{1}{K_{I}}\right)}$$

In this context, V_m_ represents the maximum reaction rate; K_m_ stands for Michaelis constant, [S] and [I] stand for the concentrations of substrate and inhibitor, respectively. The symbol v represents the initial reaction rate. K_I_ is the dissociation constant when the inhibitor binds to the free enzyme, while K_IS_ is the dissociation constant for the binding of the inhibitor to the enzyme–substrate complex.

### 2.10. Fluorescence Spectroscopy

Fluorescence spectra were recorded by mixing α-AMY (0.1 mg/mL) with varying concentrations of HF using a fluorescence spectrophotometer (F-4500, Hitachi, Tokyo, Japan). Synchronous fluorescence was measured at fixed wavelength intervals of Δλ = 15 nm (excitation 260–375 nm, emission 275 nm) and Δλ = 60 nm (excitation 220–350 nm, emission 280 nm) after 30 min incubation at 298 K. Set the excitation wavelength at 280 nm, the emission scanning wavelength range from 290 to 450 nm, the slit width at 5.0 nm, the scanning speed at 600 nm/min, and the temperature at 25 °C. Fluorescence quenching experiments were carried out at different temperatures (298, 308, and 318 K). The Stern-Volmer equation was used to analyze the fluorescence quenching mechanism (Equation (6)). The association constant (Ka) and the number of binding sites (n) were obtained through the logarithmic regression model (Equation (7)), and the thermodynamic parameters were calculated using the van’t Hoff equation (Equations (8) and (9)) [18].

(6) $$\frac{F_{0}}{F}=1+K_{\mathrm{SV}}\left[Q\right]=1+\mathrm{kq}\tau^{0}\left[Q\right]$$

(7) $$log\frac{F_{0}-\;F}{F}=\log K_{a}+\mathrm{nlog}\left[Q\right]$$

(8) $$\ln\mathrm{Ka}=\frac{-\Delta H}{\mathrm{RT}}+\frac{-\Delta S}{R}$$

(9) $$\Delta G=-\mathrm{RTIn}K_{a}=\Delta H-T\Delta S$$

where F_0_: fluorescence intensity of α-AMY; F: intensity after HF binding; K_q_: the bimolecular quenching rate constant; τ_0_: average fluorescence lifetime of α-AMY (10^−8^ s); K_sv_: Stern–Volmer quenching constant; [q]: concentration of HF; K_a_: binding constant; n: number of binding sites; R: gas constant (8.314 J·mol^−1^·K^−1^); T: absolute temperature; ΔH, ΔS, and ΔG represent enthalpy, entropy, and Gibbs free energy changes, respectively.

### 2.11. Molecular Docking

Molecular docking, a computational tool that is extensively utilized, allows for the prediction of interactions between flavonoids and proteins at the molecular scale. In order to clarify the binding mechanism between the key components of HF and α-amylase, docking simulations were carried out. The 3D crystal structure of α-amylase was obtained from the RCSB Protein Data Bank (http://www.rcsb.org/), while the ligand structures of luteolin and quercetin were retrieved from PubChem (https://pubchem.ncbi.nlm.nih.gov/). Based on a modified procedure proposed by Chen et al. [19], the interaction geometry between small molecular ligands and biomacromolecule targets was computed and characterized using Discovery Studio 2019 software.

### 2.12. Statistical Analysis

Data analysis and graphical representation were executed using Origin Pro 2021 and GraphPad Prism 10.1.2. Each set of experiments should be performed in at least three replicates, and statistical analysis was performed by one-way analysis of variance (ANOVA) followed by Duncan’s multiple range test using SPSS 26.0 (IBM, Armonk, NY, USA), and differences were considered significant at *p* < 0.05.

## 3. Results and Discussion

### 3.1. Selection and Characterization of DESs

It has been demonstrated that the physicochemical properties of DESs exert a significant influence on the extraction of flavonoids. In this experiment, six tailor-made DESs were successfully synthesized and subsequently analyzed based on six distinct types of HBDs (alcohols, acids, and sugars) with choline chloride (Chcl) serving as HBA (Table 1). Figure 2a shows the impact of various DESs, in comparison with conventional solvents (70% ethanol and water), on the extraction efficiency of total flavonoids from honeysuckle. The findings revealed that the extraction rate of flavonoids was significantly influenced by the type and inherent characteristics of DESs. It was noticeable that there were substantial disparities in the extraction rates of flavonoids among different kinds of DESs, and the DESs with hydroxyl groups demonstrated better extraction capabilities. Among the six DESs systems screened, Chcl–propylene glycol (DES-4) demonstrated the best extraction performance, with a total flavonoid yield of 8.31% ± 0.10, significantly higher than that of ethanol (4.43% ± 0.06) and water extraction (3.45% ± 0.14) (*p* < 0.05). Compared with traditional solvents, the extraction efficiency of DES-4 was approximately 1.9 times and 2.4 times higher, respectively, showing a significant advantage in the extraction of flavonoids from honeysuckle. Additionally, the Chcl–ethylene glycol (DES-5) system also exhibited a good extraction effect, with a total flavonoid yield of 6.86% ± 0.18 (*p* < 0.05), although lower than DES-4, it was still significantly better than traditional solvents. In contrast, the extraction efficacy of the combination of Chcl with malic acid, citric acid, or fructose was relatively lower. This phenomenon can be attributed to the fact that polyol molecules contain multiple hydroxyl sites, enabling them to form a denser and more continuous hydrogen bonding network with the phenolic hydroxyl and carbonyl groups of flavonoids. Such interactions enhance the solubility of flavonoids while suppressing their self-aggregation or precipitation, thereby granting polyol-based DES an advantage in dissolving and stabilizing flavonoid compounds [11]. Consistent with these findings, He et al. also reported relatively lower extraction yields when using Chcl–malic acid and Chcl–fructose systems in their study on flavonoid extraction from *Fructus aurantii*, which aligns with the results observed in the present investigation [10]. In conclusion, DES-4 demonstrated greater efficiency in extracting flavonoids from Honeysuckle compared with other synthesized DES and conventional solvents. Consequently, DES-4 was employed in the subsequent experiment.

As shown in Table 1, all prepared DES systems appeared as colorless and transparent liquids, indicating the absence of noticeable chromophoric impurities and suggesting that they are suitable as extraction media for flavonoids. The viscosity results revealed marked differences among the DES systems: DES-1, DES-2, and DES-6 exhibited relatively high viscosities (4.37 ± 0.57, 1.69 ± 0.22, and 5.01 ± 0.83 Pa·s, respectively), whereas DES-3, DES-4, and DES-5 showed much lower viscosities (0.09 ± 0.01, 0.12 ± 0.01, and 0.13 ± 0.03 Pa·s, respectively). In combination with the extraction data, it was found that the less viscous DES-4 and DES-5 generally afforded higher flavonoid yields, while the more viscous acid-type or sugar-type DESs (e.g., DES-1 and DES-6) showed relatively lower extraction efficiencies. This suggests that a moderate viscosity is beneficial for flavonoid diffusion within the solvent and for mass transfer at the solid–liquid interface, thereby enhancing overall extraction efficiency [11]. With increasing water content (up to 40%), the viscosity of all DESs decreased, particularly for DES-1 and DES-6, indicating that the addition of water may weaken intermolecular interactions and improve solution fluidity. In addition, all DESs exhibited Newtonian flow behavior over the tested shear-rate range, with only minor changes in viscosity, demonstrating good flow stability under shear conditions [20]. Overall, introducing an appropriate amount of water altered the viscosity and molecular packing of the DESs, which is expected to improve mass-transfer conditions and thus influence their extraction performance; these effects will be further examined in the subsequent single-factor experiments.

### 3.2. FT-IR Analysis

**Figure 1:**
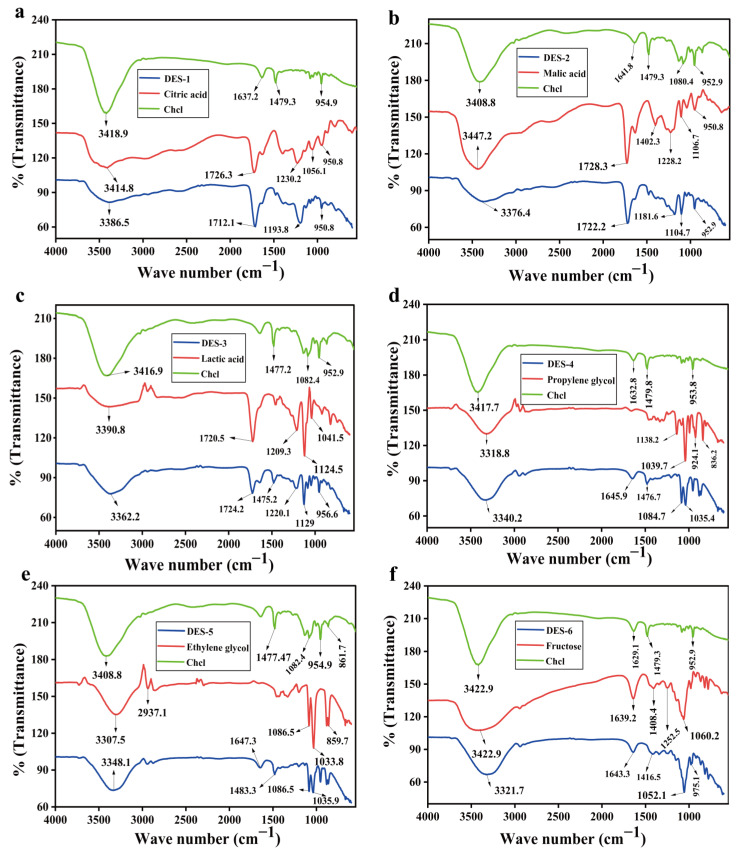
FT-IR spectroscopic characterization of prepared DESs and their individual constituents. Choline chloride (Chcl)—Citric acid (**a**); Chcl—Malic acid (**b**); Chcl—Lactic acid (**c**); Chcl—Propylene glycol (**d**); Chcl—Ethylene glycol (**e**); Chcl—Fructose (**f**).

To investigate the chemical interactions and structural changes among the components (Choline chloride, citric acid, lactic acid, malic acid, propylene glycol, ethylene glycol, fructose) and their corresponding DESs systems, this study systematically analyzed them using FT-IR. As shown in Figure 1a–f, distinct differences in FT-IR spectral features were observed among the various DES systems, which are closely related to differences in the chemical structures of HBDs, the strength of hydrogen bonding interactions, and variations in intermolecular forces [11]. Further analysis revealed that significant structural changes occurred in the FT-IR spectra of the DES systems within the ranges of 2500–3100 cm^−1^ (C–H stretching vibrations), 1416–1485 cm^−1^ (–CH_2_ bending vibrations), and 1039–1086 cm^−1^ (C–OH stretching vibrations), indicating the formation of new interactions between HBA and HBDs during DES synthesis—a characteristic feature of DES formation [21]. Particularly in the 3000–3500 cm^−1^ region, the absorption peaks of individual components were relatively sharp, suggesting weaker hydrogen bonding. In contrast, the absorption peaks in this region for the DES systems were significantly broadened, indicating a notably enhanced hydrogen bonding network. From a structure–property perspective, further analysis suggests that acid-based HBDs, due to the involvement of carboxyl groups in forming strong hydrogen bonds, exhibit more pronounced synergistic red-shifts of O–H and C=O vibrations, which may enhance solvation of polar sites such as phenolic hydroxyl groups or glycosides. Polyol-based HBDs, with multiple hydroxyl groups, facilitate the formation of continuous hydrogen bonds and enhance C–O solvation, potentially increasing affinity for polyphenols/flavonoids and facilitate their extraction [22]. In summary, the formation of a stronger hydrogen bonding network in DES systems reinforces intermolecular interactions among solvent molecules, thereby significantly improving the solubility and extraction efficiency for polar compounds.

### 3.3. Single Factor Experiment

#### 3.3.1. Ultrasonic Power

**Figure 2:**
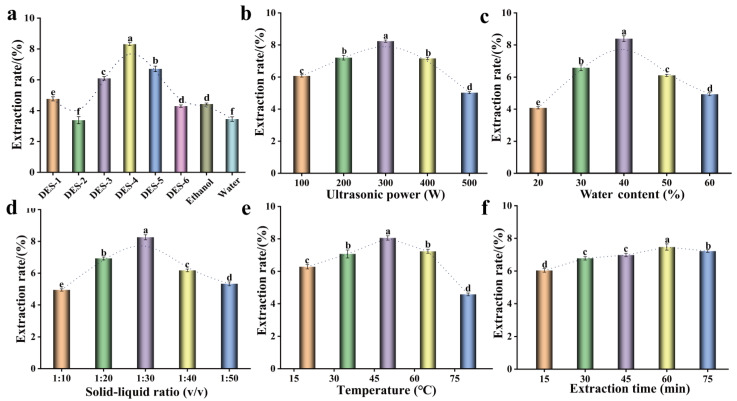
Effects of different extraction solvents on the yield of flavonoids from honeysuckle (**a**). Effects of ultrasonic power (**b**), water content (**c**), solid–liquid ratio (**d**), extraction temperature (**e**), and extraction time (**f**) on the extraction of total flavonoids from honeysuckle using DES-4-UAE. All values are the average standard deviations of three replicates. Different lowercase letters between groups indicate significant differences (*p* < 0.05).

Ultrasonic power was one of the most important factors in the ultrasound-assisted extraction process. As demonstrated in Figure 2b, the extraction efficiency of total flavonoids exhibited an initial increase followed by a subsequent decline with rising ultrasonic power, peaking at 300 W. This observation suggested that elevated ultrasonic power may enhance the cavitational effects on cellular disruption. At the same time, it can utilize the cavitation effect to produce many bubbles, hence accelerating cell rupture and aiding the dissolution of flavonoids. As the power continued to increase, the structure of some flavonoids was destroyed under the action of strong ultrasonic cavitation, and some bioactive chemicals may degrade under high ultrasonic power, resulting in a decrease in the extraction rate of total flavonoids. Previous studies have demonstrated that high ultrasound power may damage the natural active ingredients from *Polygonum aviculare leaves*, resulting in reduced yield [23]. Therefore, an ultrasonic power of 300 W was selected for the subsequent investigation.

#### 3.3.2. Water Content

As one of the limitations, the high viscosity of DESs often hinders the precipitation of target compounds, resulting in low extraction efficiency. It has been reported that the viscosity, polarity and energy transfer of DESs are significantly affected by the water content of DES [24]. The experimental results also showed that water content has a relatively large impact on the HF extraction ability. As shown in Figure 2c, as the water content in the DES system escalated from 10% to 40%, there was a marked enhancement in the extraction efficiency of flavonoids. This phenomenon can be attributed to the optimal addition of water, which reduced the viscosity of the DES, thereby facilitating a higher extraction rate. However, with a further increase in water content, a significant decline in the extraction rate of flavonoids was observed. This decline was likely due to the elevated water content within the DES system, which might hinder the interaction between the target bioactive flavonoids and the DES components. Additionally, excessive water content could disrupt the hydrogen bonding between the DES and the target compounds [5]. Studies have shown that the appropriate water content (about 42%) allows the nanostructure of DESs to be preserved, while the structure is destroyed when the water content is too high [25]. As such, the optimal water content found in this test was 40%, and this concentration was used in the subsequent condition adjustment.

#### 3.3.3. Solid–Liquid Ratio

The solid–liquid ratio plays a crucial role in the extraction efficiency of natural products. As illustrated in Figure 2d, the HF content increases significantly with the rising solid–liquid ratio. The highest extraction yield of HF is achieved at a ratio of 1:30 (g/mL). Beyond this optimum point, further increase in the solid–liquid ratio leads to a gradual decline in HF yield. This phenomenon may be attributed to the fact that an excessive solvent proportion beyond the optimal ratio necessitates additional energy input, triggering dilution effects or potentially diminishing cavitation effects, thereby reducing extraction efficiency [20]. In summary, an appropriate solvent ratio enhances interactions with plant cell structures, facilitating the extraction and dissolution of flavonoids, which consequently improves yield while reducing overall costs. Therefore, a solid–liquid ratio of 1:30 (g/mL) was selected for this experiment.

#### 3.3.4. Extract Temperature

Considering the sensitivity of flavonoids to temperature, the extraction temperature in this experiment was limited to a maximum of 80 °C. As shown in Figure 2e, the extraction rate of HF is positively correlated with temperature in the range of 20–50 °C, and the extraction efficiency drops sharply after 80 °C. Generally speaking, the increase in temperature will accelerate the movement speed of molecules, and the dissolution and diffusion rates will also accelerate accordingly. Proper heating can accelerate the solid–liquid mass transfer of solute and make flavonoids in raw materials easier to dissolve and precipitate. But when the temperature is too high, flavonoids will be oxidized and decomposed, which will destroy the structure of flavonoids and lead to a decrease in their content [26]. Some scholars used ultrasonic-assisted DESs to extract bioactive substances from blueberry leaves, and also found that too high temperature would affect the precipitation of phenolic compounds [27]. Therefore, in this experiment, 50 °C was used as the extraction temperature under the following conditions.

#### 3.3.5. Extract Time

The extraction time also affects the extraction of HF (Figure 2f). Yield increased with time, peaking at 60 min, likely due to prolonged contact between plant material and DESs facilitating flavonoid release. Beyond this point, yield declined, possibly from solvent polarity effects, prolonged ultrasonication, and temperature-induced degradation [11]. Thus, 60 min was selected as the optimal extraction time.

### 3.4. RSM Optimization

The RSM was established utilizing the findings from the single-factor experiments performed in the preceding phase. The analysis revealed three key parameters that substantially influence the extraction efficiency of flavonoids from honeysuckle: ultrasonic power (a), solid–liquid ratio (b), and water content (c). Employing the principle of Box–Behnken central composite design, 17 three-level experiments were randomly generated using the three-factor three-level RSM to assess the impact of the four independent variables and their interactions, and to identify the optimal process conditions for ultrasonic-assisted extraction of flavonoids from honeysuckle. The analysis of variance was presented in Table S2. The regression equation of each factor analyzed by fitting response surface is as follows:

$$Y\left(\%\right)\;=\;9.16432\;+\;0.3228A\;+\;0.3981B\;-\;0.2271C\;+\;0.3723\mathrm{AB}\;+\;0.4586\mathrm{AC}\;-\;0.2509\mathrm{BC}\;-\;1.92A^{2}-\;1.01B^{2}\;-\;1.25C^{2}$$

Y = yield of total flavonoids from honeysuckle (%), A = ultrasonic power (W), B is the liquid-to-solid ratio (g/mL), and C = water content (%).

As shown in Table S2, the high F-value, low *p*-value (<0.0001), and non-significant lack-of-fit term (*p* > 0.05) indicate that the model is highly significant and has good reliability. At the same time, R^2^ and high coefficients of determination (R^2^: 0.9865, Adjusted R^2^: 0.9691, Predicted R^2^: 0.9047) indicated good consistency between the model and experimental results. Therefore, this regression equation can be used to replace the actual experimental points and analyze the experimental results [28].

**Figure 3:**
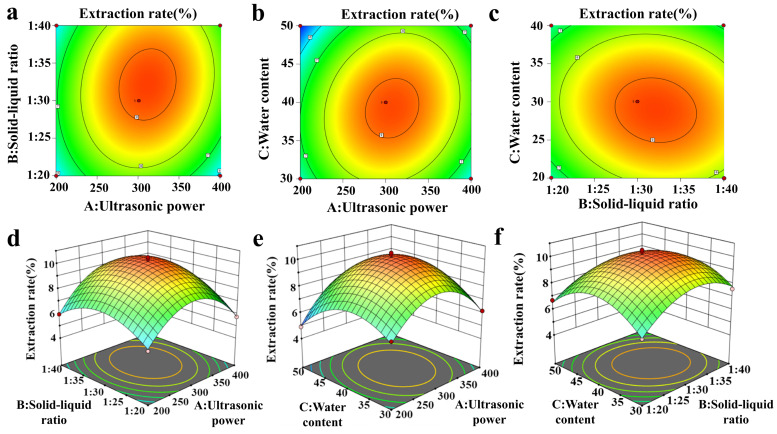
Three-dimensional response surface and contour plots of the interaction effects of different parameters on HF yield. (**a**–**c**) Contour plots showing the effects of: (**a**) ultrasonic power and solid–liquid ratio, (**b**) ultrasonic power and water content, and (**c**) solid–liquid ratio and water content on the extraction rate of total flavonoids. (**d**–**f**) 3D response surfaces showing the effects of: (**d**) ultrasonic power and solid–liquid ratio, (**e**) ultrasonic power and water content, and (**f**) solid–liquid ratio, and water content on the extraction rate of total flavonoids. Different colours indicate variations in extraction rate from low to high, the dots indicate the actual experimental point locations. (A) Ultrasonic power, (B) solid-liquid ratio, and (C) water content.

The variance analysis of the model indicates that parameters A and B, along with the quadratic terms A^2^, B^2^, and C^2^, as well as the interaction term AC, all have highly significant effects on the extraction rate of flavonoids from honeysuckle (*p* < 0.001). In contrast, the interaction term BC does not significantly affect the response value (*p* > 0.05). The interaction effect plots, 3D response surface plots and contour plots of the model show more visually whether the interaction of parameters A, B and C with each other on the total flavonoid yield of honeysuckle is significant or not, as illustrated in Figure 3a–f, the steeper and more curved the three-dimensional surface plot of the interaction term between the two factors, and the more elliptical the two-dimensional contour plot appears, the more significant the impact of the interaction term between these two factors on the response value. The steepest order of the three-dimensional figure was AC > AB > BC, indicating that the interaction between ultrasonic power and water content had the greatest influence on the response value. Moreover, the contour plots for AC and AB exhibited an elliptical shape, signifying that the interplay between ultrasonic power and water content, as well as ultrasonic power and material ratio, substantially influenced the yield of total flavonoids extracted from honeysuckle. This observation aligned with the findings from the variance model analysis. In contrast, the contour for BC appeared nearly circular, and the three-dimensional gradient was less pronounced when compared to AC and AB. This indicated that the interaction between the solid–liquid ratio and water content does not significantly affect the yield of total flavonoids from honeysuckle (*p* > 0.05).

According to the predictive model from Design Expert 13.0, the optimal extraction conditions were determined as follows: an ultrasonic power of 309 W (Because the ultrasonic apparatus allows only integer power settings, we set the power to 300 W for the validation experiments and performed multiple replicates. The extraction results closely matched the model predictions, thereby substantiating 300 W as a scientifically grounded setting), a water content of 39%, and a solid–liquid ratio of 1:32 (g/mL). Under these optimized conditions, three replicate experiments were carried out, resulting in a total flavonoid content from honeysuckle of 9.12 ± 0.08%. The experimental results closely matched the predicted values, indicating that the model demonstrates a high degree of accuracy and reliability. Compared with the research results of Li et al. [29] and Zhou et al., [30] this study achieved a higher HF extraction rate after optimization by the response surface method, indicating that this method has a significant advantage in improving the extraction efficiency of flavonoids from honeysuckle.

### 3.5. SEM Analysis

**Figure 4:**
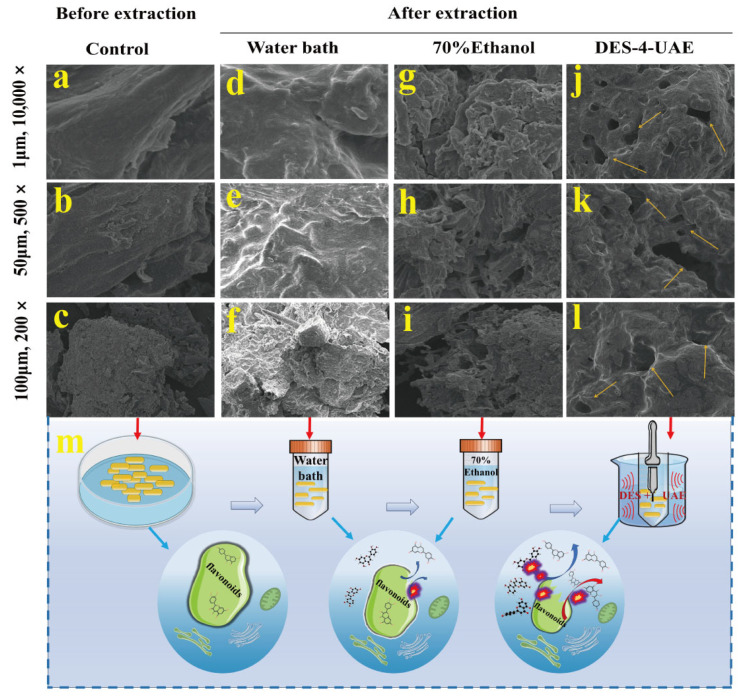
Scanning electron micrographs of honeysuckle tissue microstructure. (**a**–**l**) respectively depict the tissue surfaces of untreated, water-extracted, 70% ethanol-extracted, and DES-4-UAE-treated honeysuckle under varying magnification levels, (**m**) represents the schematic diagram of the extraction mechanism under different conditions. The yellow arrows in Figures (**j**–**l**) highlight the formation of more pronounced pores and fissures following treatment with DES-4-UAE, thereby facilitating the release of flavonoids.

Microstructural analysis of Honeysuckle tissues extracted with various solvents was performed using scanning electron microscopy. As illustrated in Figure 4a–c, the untreated material exhibited an intact and smooth initial organizational structure, with clearly larger and thicker fundamental cellular architecture on the surface. Following extraction with 70% ethanol (Figure 4d–f) and water (Figure 4g–i), the material surface displayed fine pores, cracks, and small fragments. Notably, samples treated with DES-4-UAE showed markedly weakened and atrophied surfaces, characterized by more pronounced cracking and porous structures (Figure 4j–m). These observations indicate that DES-4 may possess stronger penetrative and erosive capabilities than conventional solvents, thereby facilitating enhanced extraction of active compounds. Similar observations were reported when DESs were used to extract active compounds from *Morus alba* L. leaves, where the biomass morphology and structure were more markedly disrupted compared with other solvents [28]. In a word, DES-UAE represents a sustainable and effective method to increase the yield of bioactive compounds in plant substrates.

### 3.6. HPLC-MS Targeted Analysis and Antioxidant Activity Analysis

**Figure 5:**
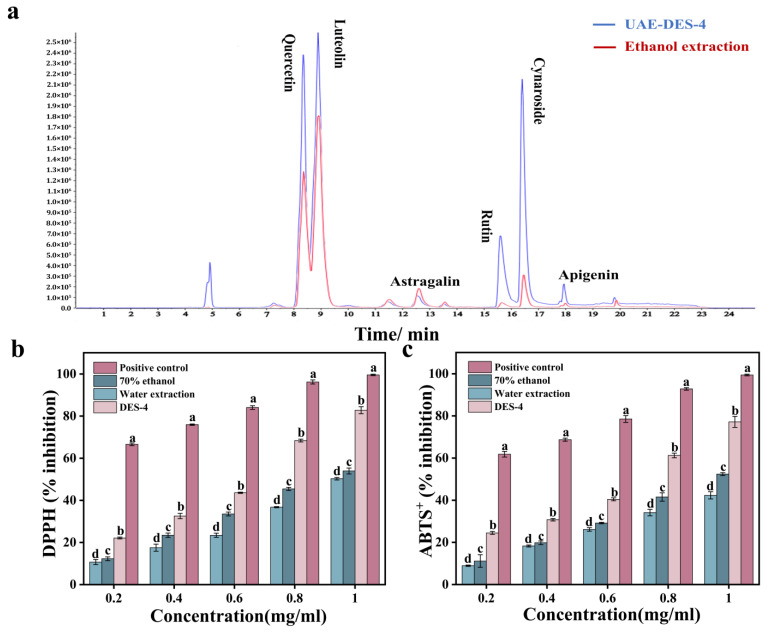
Under the optimum extraction conditions, HPLC-MS targeted analysis (DES-4-UAE and ethanol extraction) (**a**) and antioxidant activity comparison; DPPH inhibition rate (**b**); ABTS^+^ inhibition rate (**c**). All values are the average standard deviations of three replicates. Different lowercase letters between groups indicate significant differences (*p* < 0.05).

In this experiment, HF extracted under the optimal conditions was analyzed by targeted HPLC–MS and compared with extracts obtained using the conventional solvent ethanol (Figure 5a). Targeted analysis results show that UAE-DES-4 has a more significant extraction effect. The main flavonoids include luteolin, quercetin, astragalin, rutin, cynaroside, apigenin, etc., among which luteolin (4.59 ± 0.09 mg/g) and quercetin (3.05 ± 0.02 mg/g) were relatively high in HF (Table S3). Overall, DES-4-UAE was used in this experiment to achieve remarkable extraction effect, and the content of total flavonoids was significantly higher than that of ethanol extraction and some plant flowers rich in flavonoids. such as *Angelica keiskei leaves* (7.58 ± 0.35 mg/g) [31], *Eriobotrya japonica lindl* (Maximum Yield: 6.36 ±0.41 mg/g) [30]., etc. Many researchers have proven the extraction advantages of DES, for example, Ma et al. also found that DES synthesized by Chcl and lactic acid showed better extraction effect on flavonoids in *Camellia oleifera* [32].

The naturally occurring active substances, such as flavonoids, polyphenols, vitamins, and other components, present in food and medicinal plants, are considered the primary sources of natural antioxidants [33].

This experiment evaluated the free radical scavenging activities of flavonoids extracted from honeysuckle using three different solvents: DES-4-UAE, water, and 70% ethanol, along with a positive control, vitamin C, against DPPH (Figure 5b) and ABTS^+^ (Figure 5c) radicals, linear regression equation, R^2^, and IC_50_ were shown in Table S4. As illustrated, with the concentration increasing, the HF extracted by DES-4-UAE demonstrated superior antioxidant activity. The scavenging rates of DPPH and ABTS^+^ free radicals reached 82.8 ± 1.67% and 77.2 ± 2.61%, respectively, which were significantly better than those of traditional solvents (water and 70% ethanol). Moreover, the IC_50_ values of DES-4 were 0.56 and 0.60, respectively. Although slightly higher than those of the positive control, they were significantly lower than those of traditional solvents, indicating that its antioxidant effect was more prominent. Numerous studies indicate that various factors influence the mechanisms of free radical scavenging, such as solvent polarity, pH and hydrogen bonding, all of which greatly affect the overall assessment of antioxidant capacity. Results from Fourier-transform infrared spectroscopy, rheology, and scanning electron microscopy suggest that the unique properties of DES-4 may facilitate the release of flavonoid compounds, leading to higher flavonoid yields and enhanced antioxidant effects compared to traditional solvents [11]. Research by other researchers similarly confirms that the use of DES for extraction can yield higher total phenolic content and greater antioxidant potential [12,22].

### 3.7. Inhibitory Activity, Types and Kinetics of HF on α-AMY Enzyme In Vitro

α-AMY is a key digestive enzyme involved in carbohydrate hydrolysis and glucose metabolism. Its inhibition can slow carbohydrate digestion and glucose absorption, thus helping to lower postprandial blood glucose levels. Clinically, acarbose (ACA) is one of the inhibitors of α-AMY; however, its use may cause some side effects, such as gastrointestinal discomfort. Earlier investigations have shown that flavonoids, as natural compounds, may serve as effective and safe alternatives for inhibiting α-AMY [3,16,34].

**Figure 6:**
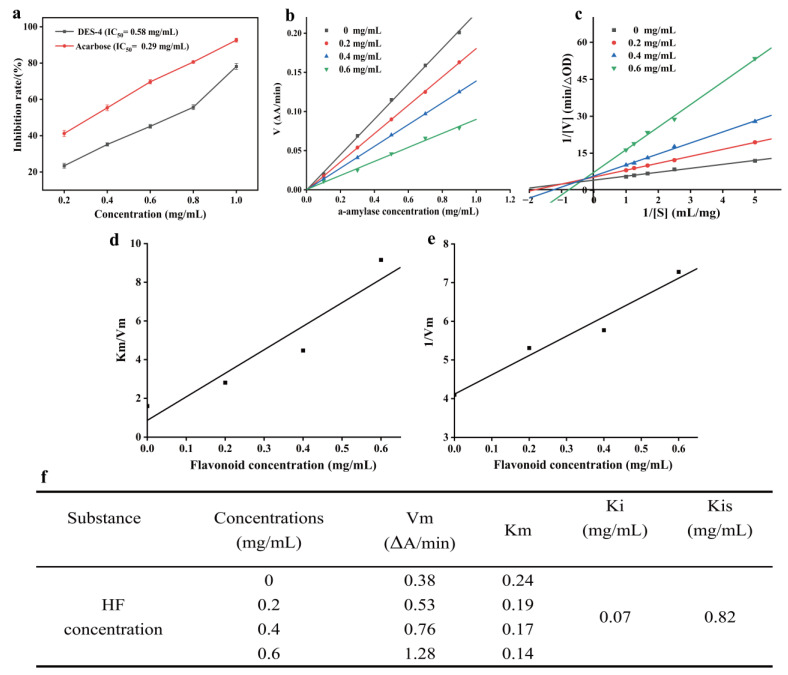
Kinetic analysis of HF-α-AMY inhibition. In vitro inhibition rate analysis (**a**), reversibility analysis (**b**), Lineweaver–Burk diagram (**c**), slope (**d**), intercept (**e**) and K_m_, V_m_, K_i_ and K_is_ values (**f**) of secondary spectrum of HF-α-AMY.

To evaluate the inhibitory activity of HF on α-AMY, Figure 6a presents the inhibitory trend of different concentrations of HF and its IC_50_ value. The results indicate that HF has a significant concentration-dependent inhibitory effect on α-AMY. Within the concentration range of 0.2–0.6 mg/mL, the inhibitory activity of HF increases significantly with the increase in concentration. When the concentration reaches 2.0 mg/mL, the inhibition rate is as high as 78.13 ± 1.65%, approaching the level of the positive control. Further calculation shows that the IC_50_ value of HF is 0.58 mg/mL, which is slightly higher than that of the positive control, acarbose. However, compared with the research results of Wang et al. [34] and Zheng et al., [35] the IC_50_ value of HF in this study is lower. This indicates that HF extracted by DES-4-UAE shows higher biological activity and hypoglycemic advantages in α-AMY inhibition. Previous studies have pointed out that HF has multiple biological functions, such as antioxidant and anti-inflammatory effects [36], these experimental results further provide new theoretical support for the use of HF as a natural hypoglycemic active component.

The reversibility of the inhibitory effect of HF on α-AMY was assessed via the relationship between enzyme concentration and the initial reaction rate (V) [37], as depicted in Figure 6b. A strong linear relationship exists between α-AMY and the reaction rate at various concentrations, and the straight line passes through the origin. Moreover, as the concentration of HF increases, the slope of the fitting equation gradually diminishes, which is consistent with the dynamic characteristics of reversible inhibition. This suggests that HF binds to α-AMY through non-covalent weak interactions and does not cause irreversible damage to the enzyme structure, thereby inhibiting its catalytic activity. This similar reversible inhibitory property was corroborated by Shen et al., in their study on the inhibition of α-AMY by hyperoside and quercetin [17].

To elucidate the specific type of HF inhibition, the Lineweaver–Burk double reciprocal method was used to analyze the enzymatic kinetic behavior. In the Lineweaver–Burk plot, the slope of the straight line represents K_m_/V_max_, while the y-intercept corresponds to 1/V_max_. Based on this, different types of enzyme inhibition can be determined [38]. As shown in Figure 6c, in the presence of HF, the linear fitting curves of 1/V versus 1/[S] intersect in the second quadrant. Moreover, in the presence of HF, the K_m_ value gradually increases from 0.38 (at 0 mg/mL) to 1.28 (at 0.6 mg/mL), while the V_max_ gradually decreases from 0.24 to 0.14, this characteristic belongs to the typical mixed-non-competitive inhibition. The calculation of the inhibition kinetic parameters reveals that the dissociation constant K_i_ of HF with the free enzyme is 0.07, whereas the dissociation constant K_is_ of HF with the enzyme–substrate complex is 0.82 (Figure 6d–f). This indicates that HF has a greater tendency to bind to the free enzyme, thereby interfering with its normal catalytic function [39]. It has been reported that certain bioflavonoids [40], coffee extract [41], chlorogenic acid and its acylated derivatives [34] also exhibit similar mixed-non-competitive inhibition characteristics. In conclusion, HF, as an effective reversible inhibitor of α-AMY, possesses potential hypoglycemic functionality, providing a theoretical foundation for the subsequent development of natural hypoglycemic functional foods.

### 3.8. Analysis of the Fluorescence Quenching Mechanism and Thermodynamic Parameters of HF on Inhibiting α-AMY

**Figure 7:**
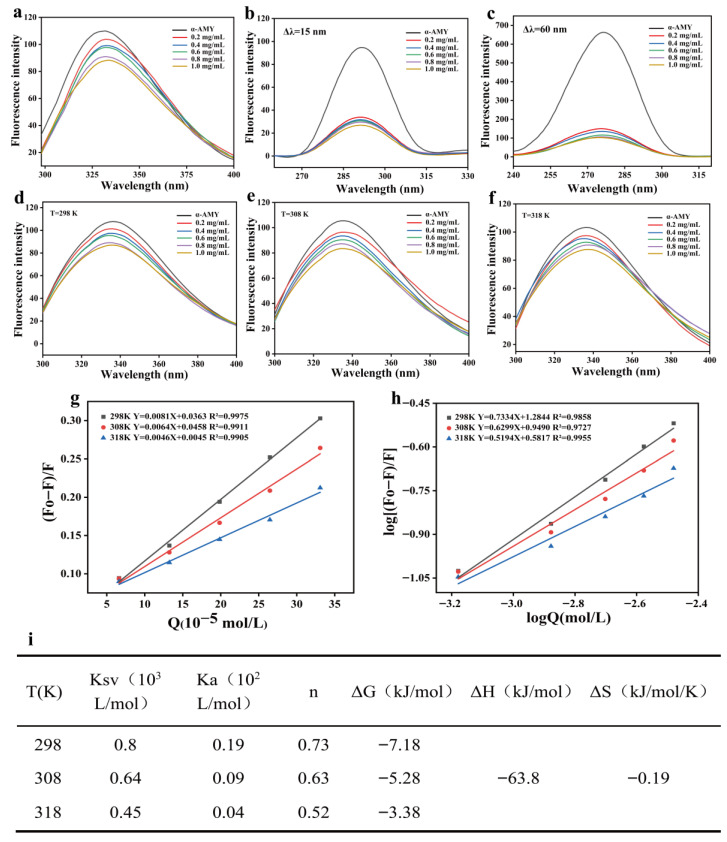
Fluorescence spectrum analysis and quenching mechanism during the interaction between HF and α-AMY. Fluorescence spectrum (**a**). In the presence of HF, the synchronous fluorescence spectra of α-AMY at 15 and 60 nm (**b**,**c**). Fluorescence spectra (**d**–**f**), The Stern–Volmer plots and logarithmic regression curves of the HF-α-AMY complex at 298 K, 308 K and 318 K, where Q represents HF concentration (**g**,**h**), the number of binding sites and the thermodynamic parameters (**i**).

The change in protein conformation can usually be evaluated by the change in the fluorescence intensity of its aromatic amino acid residues (such as Trp, Tyr and Phe) [42]. As shown in Figure 7a, with the increase in HF concentration, the fluorescence intensity of α-AMY decreased significantly, indicating that there was a concentration-dependent effect between them. This phenomenon may be related to protein conformation adjustment, energy transfer change, collision-induced quenching or the formation of ground state complex between HF and α-AMY [43]. In addition, the increase in HF concentration also causes the red shift in the maximum emission wavelength of α-AMY, suggesting that its microenvironment has changed, which may involve the exposure of hydrophobic areas or the relaxation of spatial structure. This can potentially be ascribed to the modification of the spatial conformation of α-AMY induced by the addition of HF, thereby exposing more hydrophobic regions [44].

Synchronous fluorescence spectroscopy is a sensitive approach for probing ligand-induced changes in the protein microenvironment. By simultaneously scanning excitation and emission wavelengths at fixed intervals, this method effectively monitors shifts in peak position and intensity. In this work, at 298 K, the effects of HF on α-AMY were evaluated at Δλ = 15 nm (Tyr) and Δλ = 60 nm (Trp). Results showed a concentration-dependent decrease in fluorescence intensity for both residues (Figure 7b,c). Notably, no spectral shift occurred at Δλ = 15 nm, whereas a red shift was observed at Δλ = 60 nm, suggesting that HF altered the hydrophobic environment around Trp, increasing its polarity. Similar observations were reported by Liang et al. in the inhibition of α-AMY by apigenin [45].

Fluorescence quenching is a widely adopted technique for probing ligand–protein interactions and evaluating their binding affinities. The intrinsic fluorescence of proteins primarily originates from aromatic amino acid residues, including Trp, Tyr, and Phe, and is highly sensitive to changes in the local microenvironment. In this paper, fluorescence spectroscopy was employed to assess the interaction between HF and α-AMY at varying concentrations (0.0, 0.2, 0.4, 0.6, 0.8 and 1.0 mg/mL) and temperatures (298, 308, and 318 K) (Figure 7d–f). A prominent emission peak around 330 nm was observed, mainly attributed to Trp and Tyr residues. Increasing HF concentration resulted in a marked reduction in α-AMY fluorescence intensity, suggesting conformational changes that potentially hinder energy transfer efficiency. Additionally, a slight red shift in the maximum emission wavelength indicated partial unfolding of the enzyme structure, possibly exposing hydrophobic domains and aromatic moieties to the aqueous environment. According to previous research, multispectral analysis revealed that increasing quercetin concentrations led to a marked reduction in the intrinsic fluorescence intensity of α-AMY, accompanied by a minor red shift in its maximum absorption peak [42]. Consistent with these findings, He et al. have also observed similar findings in their research on the inhibition of α-AMY by longan kernel polyphenols, which further confirms our results [38].

Fluorescence quenching typically arises from dynamic quenching, static quenching, or a combination of both mechanisms. Dynamic quenching involves energy loss through collisional interactions between the quencher and fluorophore, whereas static quenching results from the formation of a stable, non-fluorescent ground-state complex between the quencher and the fluorescent group [37].

To elucidate the inhibitory mechanism of HF on α-AMY activity, Stern–Volmer plots were generated at different temperatures. The resulting quenching curves displayed strong linearity (Figure 7g), and the Stern–Volmer constant (K_sv_) decreased progressively with rising temperature, from 0.8 × 10^2^ to 0.45 × 10^2^ L/mol. This downward trend indicates that the fluorescence quenching is primarily static in nature. Elevated temperatures may destabilize non-covalent interactions between the enzyme and ligand, thereby reducing complex stability and leading to a decline in K_sv_ [42]. The K_a_ and n values associated with static quenching were ascertained by employing a double logarithmic curve equation (Figure 7h). It was observed that K_a_ decreased as the temperature increased (Figure 7i), which implies that the formation of the HF-α-AMY complex is reversible, and the increase in temperature is not conducive to the stable existence of the HF-α-AMY complex. Moreover, the value of the binding site (n) was found to be close to 1 (Figure 7i), suggesting the presence of a potential HF binding site on α-AMY. Thermodynamic parameters, namely ΔG, ΔH, and ΔS, serve as crucial indicators for discerning non-covalent interactions, particularly those between flavonoids and proteins. The nature of the interaction force between substances is determined by ΔH and ΔS. As presented in Figure 7i, the negative values of ΔG and ΔH signify that the process of forming the HF-α-AMY complex is a spontaneous exothermic reaction, and this reaction exhibits a certain degree of randomness. Based on thermodynamic theory, a negative enthalpy change (ΔH < 0) and entropy change (ΔS < 0) indicate that hydrogen bonding is the dominant interaction; when both ΔH and ΔS are positive, hydrophobic interactions prevail; while a combination of ΔH < 0 and ΔS > 0 suggests the involvement of electrostatic forces [37]. This research, the calculated thermodynamic parameters (ΔH = −63.80 kJ/mol, ΔS = −0.19 J/mol·K) imply that the binding of HF to α-AMY is primarily governed by hydrogen bonds and van der Waals forces. Comparable findings were reported by Liang et al. who elucidated the interaction between apigenin and α-glucosidase through multi-spectral analysis [45]. Similarly, Li et al. have provided the significant contribution of hydrogen bonding and van der Waals interactions in the binding of flavonoids to α-glucosidase [40].

### 3.9. Molecular Docking Analysis

**Figure 8:**
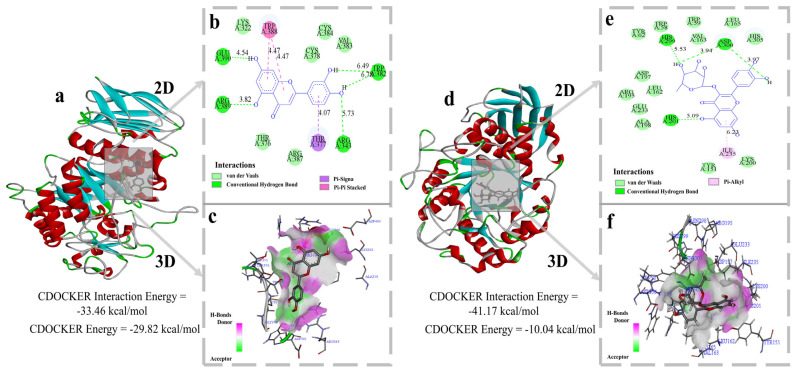
The 3D and 2D interaction diagrams of the docking of luteolin (**a**–**c**), quercetin (**d**–**f**) and α-AMY molecules. In Figures (**a**,**d**), red denotes protein α-helices, cyan denotes β-sheets, and green denotes loop regions.

To further elucidate the binding mechanism between HF and α−AMY, luteolin and quercetin−two representative flavonoids that are abundant in HF and exhibit prominent bioactivity−were selected as model ligands for molecular docking. For each ligand, multiple plausible binding poses were generated, and the complex structures of luteolin−α−AMY and quercetin−α−AMY with the lowest CDOCKER interaction energies (33.46 and 41.47 kcal/mol, respectively) were taken as the most probable binding conformations. The corresponding 3D and 2D docking results are shown in Figure 8a–f.

The docking results indicate that luteolin forms a relatively stable binding pocket on the surface of α-amylase. In its lowest-energy pose, the ligand engages in multiple hydrogen bonds with residues Glu390, Arg389, Trp382 and Arg343, with bond distances of approximately 4.54, 3.82, 6.78 and 5.73 Å, respectively. In addition, extensive van der Waals contacts are observed around Lys322, Thr376, Cys378 and Val383. Hydrophobic non-covalent interactions, including Pi-Signa and Pi-Pi Stacked, can also be detected, and these interactions collectively contribute to the spatial stability of the luteolin–α-AMY complex [46]. Quercetin exhibits a similar binding pattern. In its lowest-energy conformation, quercetin primarily forms hydrogen bonds with His299 and Asp300, van der Waals interactions with Val163, His305, Asp197 and Leu162, and hydrophobic contacts with Ile235, thereby achieving a stable association with α-AMY. These findings suggest that the representative flavonoids in HF are able to form low-energy, conformationally stable ligand–receptor complexes on the surface of α-amylase. Previous studies have shown that Asp197, Glu233 and Asp300 constitute the key catalytic residues at the active center of α-amylase, forming the “Asp–Glu–Asp” catalytic triad that is essential for its hydrolytic activity [47]. However, inspection of the lowest-energy docking poses obtained in this study reveals that luteolin and quercetin do not establish dominant direct interactions with these core catalytic residues. Instead, they preferentially bind to a neighboring, non-catalytic region that is adjacent to, but does not fully overlap with, the active site. This observation implies that the flavonoid components in HF may act as allosteric ligands that bind to peripheral sites and induce local conformational changes in the enzyme, thereby reducing the catalytic efficiency of α-amylase rather than directly occupying the active site. Such a binding mode is consistent with a non-competitive or mixed-type inhibition mechanism [17].

In terms of the nature of the intermolecular forces, hydrogen bonding and van der Waals interactions appear to be the primary driving forces for the association between HF components and α-AMY, which is in excellent agreement with the conclusions drawn from the thermodynamic analysis. Moreover, the preference of the ligands for non-catalytic regions and their potential to induce conformational rearrangements are consistent with the reversible mixed-type inhibition pattern observed in the enzyme kinetic experiments. Collectively, the molecular docking results provide strong structural and energetic support for the fluorescence quenching behavior, thermodynamic parameters and kinetic inhibition mechanism of HF toward α-amylase.

## 4. Conclusions

In this study, six DESs systems were evaluated, among which Chcl–propylene glycol (DES-4) exhibited superior performance in the extraction of flavonoids, significantly surpassing conventional solvents such as ethanol and water. Through optimization via single-factor experiments and RSM (ultrasonic power 300 W, solid–liquid ratio 1:32 (g/mL), and extraction time 60 min.), the yield of HF was markedly enhanced (9.12 ± 0.08%). HPLC-MS analysis indicated that luteolin and quercetin were the main flavonoid compounds in HF and had good antioxidant activity. The in vitro inhibition test and enzyme kinetics analysis indicated that HF had a significant inhibitory effect on α-amylase, and the inhibition mechanism was a reversible mixed-type inhibition. Fluorescence spectroscopy, thermodynamic analysis and molecular docking revealed that HF mainly bound to α-amylase through hydrogen bonds and van der Waals forces, resulting in static fluorescence quenching and changes in its hydrophobic microenvironment and spatial conformation. However, to validate in vitro findings, subsequent in vivo studies—such as investigations using diabetic animal models to assess the compound’s ability to lower postprandial blood glucose, along with bioavailability evaluations—are recommended to further characterize its biological activity and strengthen the evidence supporting its potential use in functional foods or natural medicines.

## Figures and Tables

**Table 1:** Molar ratio, abbreviation, color, viscosity and density of six natural deep eutectic solvents (Measured at 25 °C and prepared with 20/40% water). All values are the average standard deviations of three replicates. Different lowercase letters in the same column indicate significant differences (*p* < 0.05).

Composition HBA: HBD	Acronym	Molar Ratio	Water Content			
			20%		40%	
			Viscosity (Pa.s)	Density (g/cm^3^)	Viscosity (Pa.s)	Density (g/cm^3^)
Chcl: Citric acid	DES-1	1:2	4.37 ± 0.57 ^a^	1.34 ± 0.06 ^a^	0.97 ± 0.07 ^a^	1.25 ± 0.02 ^a^
Chcl: Malic acid	DES-2	1:2	1.69 ± 0.22 ^b^	1.21 ± 0.01 ^b^	0.34 ± 0.03 ^c^	1.17 ± 0.04 ^b^
Chcl: Lactic acid	DES-3	1:2	0.09 ± 0.01 ^c^	1.19 ± 0.03 ^c^	0.07 ± 0.02 ^d^	1.11 ± 0.01 ^c^
Chcl: Propylene glycol	DES-4	1:2	0.12 ± 0.01 ^c^	1.11 ± 0.02 ^e^	0.07 ± 0.01 ^d^	1.07 ± 0.03 ^d^
Chcl: Ethylene glycol	DES-5	1:2	0.13 ± 0.03 ^c^	1.09 ± 0.04 ^d^	0.09 ± 0.02 ^d^	1.01 ± 0.03 ^d^
Chcl: Fructose	DES-6	1:2	5.01 ± 0.83 ^a^	1.27 ± 0.03 ^b^	0.44 ± 0.03 ^b^	1.21 ± 0.02 ^b^

## Data Availability

The original contributions presented in the study are included in the article/Supplementary Materials, further inquiries can be directed to the corresponding author.

## Supplementary Materials

The following supporting information can be downloaded at: https://www.mdpi.com/article/10.3390/foods15010010/s1, Table S1: Level and coding of experimental design factors for response surface. Table S2: Analysis of variance. Table S3: Comparison of two main compounds in HF extracted by different solvents. Table S4: Evaluation of antioxidant activity of HF extracted with different solvents (R^2^, IC_50_).

## References

[1] Yang X., Yan D. (2025). Function, Mechanism of Action, Metabolism, and Commercial Application of *Lonicera japonica*: A Review. Food Sci. Hum. Wellness.

[2] Lin Y., Wu Y.S., Chao M., Yang D., Liu C., Tseng J., Chen Y. (2024). An Alleviative Effect of *Lonicerae japonicae* Flos Water Extract against Liver Fibrogenesis in Vitro and in Vivo. Environ. Toxicol..

[3] Sharma A.K., Sati D.M., Murti Y., Ved A., Yadav S., Singh A., Singh A., Singh M.P., Nigam A.K., Shukla K.S. (2024). A Comprehensive Review on Chinese Honeysuckle (*Qusqualis indica*): A Traditional Chinese Plant. Toxicol. Rep..

[4] Cao D., Liu Q., Jing W., Tian H., Yan H., Bi W., Jiang Y., Chen D.D.Y. (2020). Insight into the Deep Eutectic Solvent Extraction Mechanism of Flavonoids from Natural Plant. ACS Sustain. Chem. Eng..

[5] Ferreira C., Sarraguça M. (2024). A Comprehensive Review on Deep Eutectic Solvents and Its Use to Extract Bioactive Compounds of Pharmaceutical Interest. Pharmaceuticals.

[6] Bhadange Y.A., Carpenter J., Saharan V.K. (2024). A Comprehensive Review on Advanced Extraction Techniques for Retrieving Bioactive Components from Natural Sources. ACS Omega.

[7] Fu X., Belwal T., He Y., Xu Y., Li L., Luo Z. (2022). UPLC-Triple-TOF/MS Characterization of Phenolic Constituents and the Influence of Natural Deep Eutectic Solvents on Extraction of *Carya cathayensis* Sarg. Peels: Composition, Extraction Mechanism and in Vitro Biological Activities. Food Chem..

[8] Hammi K.M., Jdey A., Abdelly C., Majdoub H., Ksouri R. (2015). Optimization of Ultrasound-Assisted Extraction of Antioxidant Compounds from Tunisian Zizyphus Lotus Fruits Using Response Surface Methodology. Food Chem..

[9] Carreira-Casais A., Carpena M., Pereira A.G., Chamorro F., Soria-Lopez A., Perez P.G., Otero P., Cao H., Xiao J., Simal-Gandara J. (2021). Critical Variables Influencing the Ultrasound-Assisted Extraction of Bioactive Compounds—A Review. Chem. Proc..

[10] He Q., Tang G., Hu Y., Liu H., Tang H., Zhou Y., Deng X., Peng D., Qian Y., Guo W. (2024). Green and Highly Effective Extraction of Bioactive Flavonoids from Fructus Aurantii Employing Deep Eutectic Solvents-Based Ultrasonic-Assisted Extraction Protocol. Ultrason. Sonochem..

[11] Christou A., Parisis N.A., Venianakis T., Barbouti A., Tzakos A.G., Gerothanassis I.P., Goulas V. (2023). Ultrasound-Assisted Extraction of Taro Leaf Antioxidants Using Natural Deep Eutectic Solvents: An Eco-Friendly Strategy for the Valorization of Crop Residues. Antioxidants.

[12] Jurić T., Mićić N., Potkonjak A., Milanov D., Dodić J., Trivunović Z., Popović B.M. (2021). The Evaluation of Phenolic Content, in Vitro Antioxidant and Antibacterial Activity of Mentha Piperita Extracts Obtained by Natural Deep Eutectic Solvents. Food Chem..

[13] da Silva D.T., Pauletto R., da Silva Cavalheiro S., Bochi V.C., Rodrigues E., Weber J., da Silva C.d.B., Morisso F.D.P., Barcia M.T., Emanuelli T. (2020). Natural Deep Eutectic Solvents as a Biocompatible Tool for the Extraction of Blueberry Anthocyanins. J. Food Compos. Anal..

[14] Wang J., Zhang Y., Yu Y., Wu Z., Wang H. (2021). Combination of Ozone and Ultrasonic-Assisted Aerosolization Sanitizer as a Sanitizing Process to Disinfect Fresh-Cut Lettuce. Ultrason. Sonochemistry.

[15] Qu Y., Li C., Zhang C., Zeng R., Fu C. (2016). Optimization of Infrared-Assisted Extraction of Bletilla Striata Polysaccharides Based on Response Surface Methodology and Their Antioxidant Activities. Carbohydr. Polym..

[16] Lordan S., Smyth T.J., Soler-Vila A., Stanton C., Ross R.P. (2013). The α-Amylase and α-Glucosidase Inhibitory Effects of Irish Seaweed Extracts. Food Chem..

[17] Shen H., Wang J., Ao J., Hou Y., Xi M., Cai Y., Li M., Luo A. (2023). Structure-activity relationships and the underlying mechanism of α-amylase inhibition by hyperoside and quercetin: Multi-spectroscopy and molecular docking analyses. Spectrochim. Acta Part A Mol. Biomol. Spectrosc..

[18] Hong J., Choi Y., Lee J., Park Y.J., Lee D.Y., Chang P.-S. (2023). Inhibitory Characteristics of Flavonoids from Soybean (*Glycine max* [L.] Merr.) Leaf against Pancreatic Lipase. Food Biosci..

[19] Chen Z., Liu W., Fu M., Xu Y., Chen J., Geng Q., Li T., Dai T. (2026). Impact of Glycosylation Position on Functional Characteristics and Interaction Mechanisms in Pea Protein-Flavonoid Complexes: A Comparative Study of Genistein and Its O-Glycosides. Food Hydrocoll..

[20] Rashid R., Mohd Wani S., Manzoor S., Masoodi F.A., Masarat Dar M. (2023). Green Extraction of Bioactive Compounds from Apple Pomace by Ultrasound Assisted Natural Deep Eutectic Solvent Extraction: Optimisation, Comparison and Bioactivity. Food Chem..

[21] Al-Risheq D.I.M., Nasser M.S., Qiblawey H., Hussein I.A., Benamor A. (2021). Choline Chloride Based Natural Deep Eutectic Solvent for Destabilization and Separation of Stable Colloidal Dispersions. Sep. Purif. Technol..

[22] Ozturk B., Parkinson C., Gonzalez-Miquel M. (2018). Extraction of Polyphenolic Antioxidants from Orange Peel Waste Using Deep Eutectic Solvents. Sep. Purif. Technol..

[23] Wu L., Chen Z., Li S., Wang L., Zhang J. (2021). Eco-Friendly and High-Efficient Extraction of Natural Antioxidants from Polygonum Aviculare Leaves Using Tailor-Made Deep Eutectic Solvents as Extractants. Sep. Purif. Technol..

[24] Shang X., Chu D., Zhang J., Zheng Y., Li Y. (2021). Microwave-Assisted Extraction, Partial Purification and Biological Activity in Vitro of Polysaccharides from Bladder-Wrack (*Fucus vesiculosus*) by Using Deep Eutectic Solvents. Sep. Purif. Technol..

[25] Hammond O.S., Bowron D.T., Edler K.J. (2017). The Effect of Water upon Deep Eutectic Solvent Nanostructure: An Unusual Transition from Ionic Mixture to Aqueous Solution. Angew. Chem..

[26] Chu G., Liang R., Wan C., Yang J., Li J., Wang R., Du L., Lin R. (2022). Ultrasonic-Assisted Extraction of Flavonoids from *Juglans mandshurica* Maxim.: Artificial Intelligence-Based Optimization, Kinetics Estimation, and Antioxidant Potential. Molecules.

[27] Santos-Martín M., Cubero-Cardoso J., González-Domínguez R., Cortés-Triviño E., Sayago A., Urbano J., Fernández-Recamales Á. (2023). Ultrasound-Assisted Extraction of Phenolic Compounds from Blueberry Leaves Using Natural Deep Eutectic Solvents (NADES) for the Valorization of Agrifood Wastes. Biomass Bioenergy.

[28] Zhou P., Wang X., Liu P., Huang J., Wang C., Pan M., Kuang Z. (2018). Enhanced Phenolic Compounds Extraction from *Morus alba* L. Leaves by Deep Eutectic Solvents Combined with Ultrasonic-Assisted Extraction. Ind. Crop. Prod..

[29] Li J., Wu C., Li F., Yu R., Wu X., Shen L., Liu Y., Zeng W. (2019). Optimization of Ultrasound-Assisted Water Extraction of Flavonoids from *Psidium guajava* Leaves by Response Surface Analysis. Prep. Biochem. Biotechnol..

[30] Zhou C., Sun C., Chen K., Li X. (2011). Flavonoids, Phenolics, and Antioxidant Capacity in the Flower of *Eriobotrya japonica* Lindl. Int. J. Mol. Sci..

[31] Zhang W., Jin Q., Luo J., Wu J., Wang Z. (2018). Phytonutrient and Anti-Diabetic Functional Properties of Flavonoid-Rich Ethanol Extract from *Angelica Keiskei* Leaves. J. Food Sci. Technol..

[32] Ma Y., Liu M., Tan T., Yan A., Guo L., Jiang K., Tan C., Wan Y. (2018). Deep Eutectic Solvents Used as Extraction Solvent for the Determination of Flavonoids from *Camellia oleifera* Flowers by High-Performance Liquid Chromatography. Phytochem. Anal. PCA.

[33] Xu D.-P., Li Y., Meng X., Zhou T., Zhou Y., Zheng J., Zhang J.-J., Li H.-B. (2017). Natural Antioxidants in Foods and Medicinal Plants: Extraction, Assessment and Resources. Int. J. Mol. Sci..

[34] Wang S., Li Y., Huang D., Chen S., Xia Y., Zhu S. (2022). The Inhibitory Mechanism of Chlorogenic Acid and Its Acylated Derivatives on α-Amylase and α-Glucosidase. Food Chem..

[35] Zheng Y., Tian J., Yang W., Chen S., Liu D., Fang H., Zhang H., Ye X. (2020). Inhibition Mechanism of Ferulic Acid against α-Amylase and α-Glucosidase. Food Chem..

[36] Li Y., Li W., Fu C., Song Y., Fu Q. (2019). Lonicerae Japonicae Flos and Lonicerae Flos: A Systematic Review of Ethnopharmacology, Phytochemistry and Pharmacology. Phytochem. Rev..

[37] Yang J., Wang X., Zhang C., Ma L., Wei T., Zhao Y., Peng X. (2021). Comparative Study of Inhibition Mechanisms of Structurally Different Flavonoid Compounds on α-Glucosidase and Synergistic Effect with Acarbose. Food Chem..

[38] He T., Zhao L., Chen Y., Zhang X., Hu Z., Wang K. (2021). Longan Seed Polyphenols Inhibit α-Amylase Activity and Reduce Postprandial Glycemic Response in Mice. Food Funct..

[39] Thayumanavan P., Nallaiyan S., Loganathan C., Sakayanathan P., Kandasamy S., Isa M.A. (2020). Inhibition of Glutathione and S-Allyl Glutathione on Pancreatic Lipase: Analysis through in Vitro Kinetics, Fluorescence Spectroscopy and in Silico Docking. Int. J. Biol. Macromol..

[40] Li H., Yang J., Wang M., Ma X., Peng X. (2023). Studies on the Inhibition of α-Glucosidase by Biflavonoids and Their Interaction Mechanisms. Food Chem..

[41] Li X., Cai J., Yu J., Wang S., Copeland L., Wang S. (2021). Inhibition of in Vitro Enzymatic Starch Digestion by Coffee Extract. Food Chem..

[42] Huang M., Xiao Q., Li Y., Ahmad M., Tang J., Liao Q., Tan C. (2024). Inhibition of α-Amylase Activity by Quercetin via Multi-Spectroscopic and Molecular Docking Approaches. Food Biosci..

[43] Joye I.J., Davidov-Pardo G., Ludescher R.D., McClements D.J. (2015). Fluorescence Quenching Study of Resveratrol Binding to Zein and Gliadin: Towards a More Rational Approach to Resveratrol Encapsulation Using Water-Insoluble Proteins. Food Chem..

[44] Wang S., Xie X., Zhang L., Hu Y., Wang H., Tu Z. (2020). Inhibition Mechanism of α-Glucosidase Inhibitors Screened from *Artemisia selengensis* Turcz Root. Ind. Crop. Prod..

[45] Liang F., Meng K., Pu X., Cao Y., Shi Y., Shi J. (2024). Deciphering the Binding Behavior and Interaction Mechanism of Apigenin and α-Glucosidase Based on Multi-Spectroscopic and Molecular Simulation Studies. Int. J. Biol. Macromol..

[46] Chen Z., Fu M., Chen J., Zhang G., Li T., Geng Q., Dai T. (2025). Study on the Interaction Mechanism and Physicochemical Properties of Luteolin and Its Glucoside Compounds with β-Lactoglobulin. LWT.

[47] Ramakrishnan K., Rajan R., Nachimuthu L., Jayaraj P., Narasimhulu C.A., Deme P., Rajagopalan S., Sivaramakrishna A., Karthikeyan S., Desikan R. (2025). Development of Novel α-Amylase Inhibitors: Synthesis, Molecular Docking, and Biochemical Studies. Cell Biochem. Biophys..

