# Supplementary material for: Ultrasound-Assisted Deep Eutectic Solvent-Based Green Extraction of Flavonoids from Honeysuckle: Optimization and Mechanistic Insights into α-Amylase Inhibition
Source: Foods. 2025 Dec 19;15(1):10. doi: 10.3390/foods15010010 (PMC12786239; doi:10.3390/foods15010010)
Supplement: Supplementary file 1 [file foods-15-00010-s001.zip › Table S1.pdf]

Table S1

Level and coding of experimental design factors for response surface

Sequence	A Ultrasonic power /W	B Solid-liquid ratio/ (g/ mL)	C Moisture content /%	yield /%
1	300	40	30	7.59
2	200	20	40	5.76
3	400	30	50	6.38
4	200	30	30	6.52
5	200	30	50	4.92
6	400	40	40	7.45
7	300	30	40	9.36
8	400	30	30	6.16
9	300	30	40	9.01
10	300	30	40	9.50
11	200	40	40	5.96
12	300	20	50	6.72
13	300	30	40	8.83
14	400	20	40	5.76
15	300	40	50	6.87
16	300	20	30	6.44
17	300	30	40	9.12
