# Supplementary material for: Ultrasound-Assisted Deep Eutectic Solvent-Based Green Extraction of Flavonoids from Honeysuckle: Optimization and Mechanistic Insights into α-Amylase Inhibition
Source: Foods. 2025 Dec 19;15(1):10. doi: 10.3390/foods15010010 (PMC12786239; doi:10.3390/foods15010010)
Supplement: Supplementary file 1 [file foods-15-00010-s001.zip › Table S2.pdf]

Table S2

Analysis of variance

Source	Sum of Squares	df	Mean Square	F-value	p-value	Significance
Model	33.36	9	3.71	56.74	< 0.0001	significant
A	0.83	1	0.83	12.76	0.0091	
B	1.27	1	1.27	19.41	0.0031	
C	0.41	1	0.41	6.32	0.0402	
AB	0.55	1	0.55	8.49	0.0225	
AC	0.84	1	0.84	12.88	0.0089	
BC	0.25	1	0.25	3.85	0.0904	
A^2	15.51	1	15.51	237.44	< 0.0001	
B^2	4.31	1	4.31	66.00	< 0.0001	
C^2	6.58	1	6.58	100.74	< 0.0001	
Residual	0.46	7	0.07			
Lack of Fit	0.17	3	0.06	0.82	0.55	not significant
Pure Error	0.28	4	0.07			
Cor Total	33.81		16			
R^2 = 0.9865	R^2 Adj = 0.9691		R^2 Pre = 0.9047			
