# Supplementary material for: Ultrasound-Assisted Deep Eutectic Solvent-Based Green Extraction of Flavonoids from Honeysuckle: Optimization and Mechanistic Insights into α-Amylase Inhibition
Source: Foods. 2025 Dec 19;15(1):10. doi: 10.3390/foods15010010 (PMC12786239; doi:10.3390/foods15010010)
Supplement: Supplementary file 1 [file foods-15-00010-s001.zip › Table S3.pdf]

Table S3

Comparison of two main compounds in HF extracted by different solvents.

Compound	Linear equation	R2	LOQ	RT (min)	DES-4-UAE content（mg/g）	Ethanol extraction content（mg/g）
Quercetin	y = 8.41541e4 x + 7.60471e4	0.99533	1	15.64	3.05 ± 0.02	2.15 ± 0.04
Luteolin	y = 8.41541e4 x + 7.60471e4	0.99149	1	16.42	4.59 ± 0.09	3.76 ± 0.07
