# Supplementary material for: Ultrasound-Assisted Deep Eutectic Solvent-Based Green Extraction of Flavonoids from Honeysuckle: Optimization and Mechanistic Insights into α-Amylase Inhibition
Source: Foods. 2025 Dec 19;15(1):10. doi: 10.3390/foods15010010 (PMC12786239; doi:10.3390/foods15010010)
Supplement: Supplementary file 1 [file foods-15-00010-s001.zip › Table S4.pdf]

Table S4

Evaluation of antioxidant activity of HF extracted with different solvents (R^2, IC_{50}).

Sample	DPPH			$ABTS^+$		
	Linear regression equation	R^2	IC_{50} (mg /mL)	Linear regression equation	R^2	IC_{50} (mg /mL)
Positive control	Y = 43.10X + 58.60	0.9803	0.1282	Y = 49.62X + 50.50	0.9849	0.154
DES-4	Y = 78.58X + 2.75	0.9741	0.5570	Y = 67.90X + 6.08	0.9578	0.604
70% ethanol	Y = 52.65X + 2.15	0.9979	0.920	Y = 52.12X - 0.46	0.9977	0.990
Water	Y = 49.22X - 1.78	0.9655	1.079	Y = 41.32X + 1.18	0.9990	1.310
